# Selection criteria of residents for residency programs in Kuwait

**DOI:** 10.1186/1472-6920-13-4

**Published:** 2013-01-19

**Authors:** Yousef Marwan, Adel Ayed

**Affiliations:** 1 Department of Surgery and Centre for Medical Education, Faculty of Medicine, Health Sciences Center, Kuwait University, Al-Jabriya, Kuwait

**Keywords:** Residents, Selection, Criteria, Residency, Postgraduate training, Medical education, Kuwait

## Abstract

**Background:**

In Kuwait, 21 residency training programs were offered in the year 2011; however, no data is available regarding the criteria of selecting residents for these programs. This study aims to provide information about the importance of these criteria.

**Methods:**

A self-administered questionnaire was used to collect data from members (e.g. chairmen, directors, assistants …etc.) of residency programs in Kuwait. A total of 108 members were invited to participate. They were asked to rate the importance level (scale from 1 to 5) of criteria that may affect the acceptance of an applicant to their residency programs. Average scores were calculated for each criterion.

**Results:**

Of the 108 members invited to participate, only 12 (11.1%) declined to participate. Interview performance was ranked as the most important criteria for selecting residents (average score: 4.63/5.00), followed by grade point average (average score: 3.78/5.00) and honors during medical school (average score: 3.67/5.00). On the other hand, receiving disciplinary action during medical school and failure in a required clerkship were considered as the most concerning among other criteria used to reject applicants (average scores: 3.83/5.00 and 3.54/5.00 respectively). Minor differences regarding the importance level of each criterion were noted across different programs.

**Conclusions:**

This study provided general information about the criteria that are used to accept/reject applicants to residency programs in Kuwait. Future studies should be conducted to investigate each criterion individually, and to assess if these criteria are related to residents' success during their training.

## Background

The process of applying to a residency training program is stressful for students and junior doctors. Thus, information about variables that may affect the acceptance of an applicant into his/her preferred program were provided in the literature. Green et al. (2009) and Wagoner and Suriano (1999) surveyed directors of residency programs in the United States of America (USA) in order to determine the selection criteria of residents [1,2]. Both studies revealed that the applicant's grades in required clerkships, number of honors during medical school and United States Medical Licensing Examination (USMLE) step 1 score are the most important criteria. They also revealed that some selection criteria differ in their importance level across different programs according to their competitiveness level, for example published medical school research was ranked higher by directors of competitive programs. On the other hand, consultants in the United Kingdom (UK) considered previous specialty-specific experience gained during foundation (intern-level) training and additional research degrees as the most important criteria in selecting residents for residency training [3]. Moreover, several studies explored the criteria of selecting residents within specific specialties [4-12], while others assessed the importance of each criterion individually [13-16].

The literature lacks studies that provide information about the criteria of selecting residents for residency programs in the Middle East. In Kuwait, Kuwait Institute for Medical Specialization (KIMS) developed one residency program (Family Medicine) in the year 1983. During 2011, 21 residency programs were offered by KIMS; however, no data were available regarding the criteria of selecting residents. By conducting this study, we intended to assess the importance level, as perceived by residency programs' members, of criteria that may affect the selection of residents for residency programs in Kuwait. We aimed to provide a guide for medical students and junior doctors who are interested in applying for these residency training programs.

## Methods

After a thorough review of the literature and based on our knowledge about the process of selecting residents in Kuwait, we developed a self-administered questionnaire in English containing 24 criteria that may affect the selection of residents to residency programs in Kuwait. These criteria were divided into 16 positive (increase the chance of acceptance) and 8 negative (lower the chance of acceptance) criteria. The participants were asked to rate these criteria. The rating scale of the positive criteria was as follows: 5 = critical; if present usually guarantee selecting the applicant, 4 = important; if present is very useful in selecting the applicant, 3 = not very important, but useful if present, 2 = rarely considered when selecting residents, 1 = not important at all when selecting residents. On the other hand, the rating scale of the negative criteria was as follows: 5 = critically concerning; if present usually guarantee rejecting the applicant, 4 = very concerning; if present is likely used to reject the applicant, 3 = not very concerning; may be considered to reject applicants if there is a strong competition between applicants, 2 = rarely considered as concerning; rarely used to reject applicants, 1 = not a concern; not used at all in rejecting applicants. The questionnaire was pretested on 6 members of general surgery residency program to ensure clarity of the questions. Moreover, data regarding residency programs in Kuwait (i.e. offered programs, number of applicants and number of residents accepted) were obtained from KIMS.

This cross-sectional study was conducted during the period between August and December 2011. The study sample consisted of members (i.e. chairmen, directors, assistants and coordinators) of all residency programs in Kuwait who already interviewed applicants during March 2011. One hundred and eight members were invited to participate.

The study protocol and data collection instrument was approved by the Joint Ethics Committee of the Ministry of Health in Kuwait and the Faculty of Medicine in Kuwait University. A written informed consent was obtained from each participant after a clear explanation of the objectives of the study. Also, the participants were assured the confidentiality of the collected information and that they were free to decline participation in the study.

Data were analyzed using the Statistical Package for Social Sciences (SPSS). We measured the overall average score of each item to rank the criteria of selecting residents. In addition, in order to compare between each program, we considered the average score for each item within each specialty during our analyses.

## Results

Out of the 108 members of residency programs invited to participate, only 12 (11.1%) declined to participate in the study without reporting any reason for their decline. According to the seats offered and number of applicants for each program, Emergency Medicine, Radiology and Dermatology are the most competitive programs to get accepted in, while some of Laboratory Medicine programs (Clinical Biochemistry, Clinical Virology, and Histo- and Cyto- Pathology) are the least competitive (Table 1). Nevertheless, all residency programs, except Primary Health Care and Histo- and Cyto- Pathology, accepted less than 50% of their total number of applicants in 2011.

**Table 1:** Frequencies of applicants for residency programs in Kuwait, 2011

**Program**	**Applied‡**				**Accepted**			
	Opt 1*	**Opt 2**	**Opt 3**	**Total**	R1*	**R2**	**R3**	**Total**
Anesthesia	43 (89.6%)	2 (4.2%)	3 (6.25%)	48 (100.0%)	10 (20.8%)	6 (12.5%)	7 (14.6%)	23 (47.9%)
Dermatology	46 (59.7%)	22 (28.6%)	9 (11.7%)	77 (100.0%)	8 (10.4%)	0 (0.0%)	0 (0.0%)	8 (10.4%)
Emergency Medicine	108 (85.0%)	10 (7.9%)	9 (7.1%)	127 (100.0%)	4 (3.2%)	0 (0.0%)	0 (0.0%)	4 (3.2%)
General Surgery	23 (62.2%)	8 (21.6%)	6 (16.2%)	37 (100.0%)	11 (29.7%)	0 (0.0%)	0 (0.0%)	11 (29.7%)
Internal Medicine	51 (67.1%)	19 (25.0%)	6 (7.9%)	76 (100.0%)	28 (36.8%)	2 (2.6%)	0 (0.0%)	30 (39.5%)
Laboratory-Clinical Biochemistry & Metabolism	1 (8.3%)	6 (50%)	5 (41.7%)	12 (100.0%)	3 (25.0%)	0 (0.0%)	0 (0.0%)	3 (25.0%)
Laboratory-Clinical Hematology	9 (34.6%)	10 (38.5%)	7 (26.9%)	26 (100.0%)	5 (19.2%)	0 (0.0%)	1 (3.8%)	6 (23.1%)
Laboratory-Clinical Microbiology	10 (50.0%)	6 (30.0%)	4 (20.05)	20 (100.0%)	10 (50.0%)	0 (0.0%)	0 (0.0%)	10 (50.0%)
Laboratory-Clinical Virology	1 (10.0%)	4 (40.0%)	5 (50.0%)	10 (100.0%)	3 (30.0%)	0 (0.0%)	0 (0.0%)	3 (30.0%)
Laboratory-Diagnostic Immunology	6 (42.8%)	0 (0.0%0	8 (57.1%)	14 (100.0%)	2 (14.3%)	0 (0.0%)	0 (0.0%)	2 (14.3%)
Laboratory-(Histo- & Cyto-) Pathology	0 (0.0%)	4 (66.7%)	2 (33.3%)	6 (100.0%)	4 (66.7%)	0 (0.0%)	0 (0.0%)	4 (66.7%)
Nuclear Medicine	14 (51.8%)	10 (37.0%)	3 (11.1%)	27 (100.0%)	4 (14.8%)	0 (0.0%)	0 (0.0%)	4 (14.8%)
Obstetrics & Gynecology	43 (95.6%)	1 (2.2%)	1 (2.2%)	45 (100.0%)	10 (22.2%)	3 (6.7%)	2 (4.4%)	15 (33.3%)
Ophthalmology	15 (62.5%)	2 (8.3%)	7 (29.2%)	24 (100.0%)	10 (41.7%)	0 (0.0%)	0 (0.0%)	10 (41.7%)
Orthopedic Surgery	11 (47.8%)	8 (34.8%)	4 (17.4%)	23 (100.0%)	8 (34.8%)	0 (0.0%)	1 (4.3%)	9 (39.1%)
Otorhinolaryngology (Head & Neck Surgery)	17 (36.2%)	18 (38.3%)	12 (25.5%)	47 (100.0%)	3 (6.4%)	0 (0.0%)	0 (0.0%)	3 (6.4%)
Pediatrics	18 (60.0%)	9 (30.0%)	3 (10.0%)	30 (100.0%)	8 (26.7%)	0 (0.0%)	0 (0.0%)	8 (26.7%)
Physical Medicine & Rehabilitation	6 (100.0%)	0 (0.0%)	0 (0.0%)	6 (100.0%)	2 (33.3%)	0 (0.0%)	0 (0.0%)	2 (33.3%)
Primary Health Care	35 (40.2%)	32 (36.8%)	20 (23.0%)	87 (100.0%)	45 (51.7%)	0 (0.0%)	0 (0.0%)	45 (51.7%)
Radiology	63 (63.0%)	21 (21.0%)	16 (16.0%)	100 (100.0%)	12 (12.0%)	4 (4.0%)	0 (0.0%)	16 (16.0%)
Urology	14 (60.9%)	7 (30.4%)	2 (8.7%)	23 (100.0%)	6 (26.1%)	0 (0.0%)	0 (0.0%)	6 (26.1%)

*Opt = Option; R = Residency year.

‡Applicants have the right to apply for 3 different residency training programs and decide their preference from 1 to 3.

Table 2 and Table 3 demonstrate the criteria that may positively and negatively affect selecting residents for residency programs in Kuwait respectively. Interview performance was ranked as the most important criteria used in selecting residents (average score = 4.63/5.00), while receiving disciplinary actions during medical school was ranked as the most important criteria used in rejecting residents (average score = 3.83/5.00). On the other hand, grades in pre-clinical courses, meaningful involvement in extracurricular activities during medical school and gender of the applicant were considered not important when selecting residents (average scores = 2.98/5.00, 2.87/5.00 and 1.71/5.00 respectively). Likewise, graduating in the lower third of the class, receiving a failure in a pre-clinical course, having family responsibilities and not participating in extra-curricular activities during medical were rarely considered as concerning when selecting residents (average scores = 2.98/5.00, 2.84/5.00, 2.27/5.00, 2.16/5.00 respectively).

**Table 2:** Average score of criteria that may positively affect the acceptance of an applicant to residency programs in Kuwait, 2011

**Rank**	**Criteria**	Score*
1	Interview performance	4.63
2	Grade Point Average (GPA)	3.78
3	Honors during medical school	3.67
4	Nationality	3.59
5	Grades in required clerkships	3.43
6/7	Electives in required clerkships	3.41
6/7	Research experience	3.41
8/9	Medical school reputation	3.39
8/9	Recommendation letters	3.39
10	Grades in clinical courses	3.37
11	Publications in indexed journals	3.33
12	Rank on class during medical school	3.27
13	Attending scientific conferences during medical school	3.15
14	Grades in pre-clinical courses	2.98
15	Meaningful involvement in extracurricular activities during medical school	2.87
16	Gender	1.71

*Scoring scale:

5 = critical; if present usually guarantee selecting the applicant.

4 = important; if present is very useful in selecting the applicant.

3 = not very important, but useful if present.

2 = rarely considered when selecting residents.

1 = not important at all when selecting residents.

**Table 3:** Average score of criteria that may negatively affect the acceptance of an applicant to residency programs in Kuwait, 2011

**Rank**	**Criteria**	Score*
1	Received disciplinary action in medical school	3.83
2	Received failure in a required clerkship	3.54
3	Took extended time to graduate for academic reasons	3.47
4	Spent a long period after internship to apply for a residency program	3.10
5	Graduated in the lower third of class	2.98
6	Received a failure in a preclinical course	2.84
7	Had family responsibilities	2.27
8	Did not participate in extracurricular activities during medical school	2.16

*Scoring scale:

5 = critically concerning; if present usually guarantee rejecting the applicant.

4 = very concerning; if present is likely used to reject the applicant.

3 = not very concerning; may be considered to reject applicants if there is a strong competition between applicants.

2 = rarely considered as concerning; rarely used to reject applicants.

1 = not a concern; not used at all in rejecting applicants.

Tables 4, 5 and 6 demonstrate the average scores of the criteria of selecting residents within each specialty individually. Minor differences existed between specialties. Interview performance was the most important criterion across all programs except for Urology where nationality was more important in selecting residents, and Otorhinolaryngology were electives in the required clerkship and research experience were also more important than the interview. Members of Anesthesia program considered medical school reputation as important as interview performance. Also, nationality was considered as important as interview performance by Clinical Virology and Nuclear Medicine programs' members. On the other hand, the most important criteria used in rejecting applicants to residency programs are receiving disciplinary actions in medical school, receiving failure in the required clerkship and spending a long period to graduate from medical school due to academic reasons; however, spending a long period after internship to apply for a residency program was considered as the most concerning criterion by Emergency Medicine program members. Moreover, members of Ophthalmology residency program considered graduating in the lower third of the class as concerning as spending a long time to graduate from medical school due to academic reasons.

**Table 4:** Average score* of criteria that may affect the acceptance of an applicant to Pediatrics and Medical residency programs in Kuwait, 2011

**Criteria**	**Dermatology**	**Emergency Medicine**	**Internal Medicine**	**Pediatrics**	**Physical Medicine & Rehabilitation**	**Primary Health Care**
	**N = 3**	**N = 4**	**N = 8**	**N = 6**	**N = 3**	**N = 4**
Positive factors						
Gender	2.66	1.50	1.00	1.33	1.66	3.00
Nationality	4.33	3.25	3.62	3.66	4.33	3.00
Grade Point Average (GPA)	4.33	1.75	4.00	3.00	3.66	3.25
Honors during medical school	4.00	2.25	4.00	3.00	3.00	3.25
Rank on class during medical school	4.00	1.50	3.25	2.83	3.33	2.00
Grades in required clerkships	4.66	1.75	3.62	3.33	3.66	2.25
Grades in clinical courses	4.33	1.75	3.25	4.00	4.00	2.25
Grades in pre-clinical courses	3.66	1.50	2.62	3.16	3.66	2.25
Electives in required clerkships	3.33	2.50	3.25	3.16	3.66	1.75
Research experience	3.66	2.75	3.50	3.00	4.00	2.50
Publications in indexed journals	3.66	2.25	3.87	3.16	3.33	2.25
Recommendation letters	4.00	2.50	3.37	3.66	3.66	3.50
Attending scientific conferences during medical school	4.00	1.75	3.50	3.83	3.33	2.00
Medical school reputation	3.66	1.75	3.75	3.50	3.33	3.50
Meaningful involvement in extracurricular activities during medical school	3.00	2.00	2.87	3.00	3.00	2.25
Interview performance	5.00	4.75	4.25	4.66	4.66	4.50
Negative factors						
Received disciplinary action in medical school	4.66	2.50	4.25	2.83	4.00	3.25
Received failure in a required clerkship	4.66	2.75	3.37	3.50	3.66	2.75
Took extended time to graduate for academic reasons	4.33	2.25	3.87	2.33	2.66	3.50
Graduated in the lower third of class	4.00	1.50	3.12	2.33	2.66	2.50
Received a failure in a preclinical course	3.66	1.50	2.87	2.66	2.66	2.75
Had family responsibilities	2.33	2.50	1.87	2.00	2.00	3.25
Spent a long period after internship to apply for a residency program	2.66	3.25	3.37	2.50	2.66	2.75
Did not participate in extracurricular activities during medical school	2.00	1.50	1.75	2.16	1.66	1.50

*Scoring scale (positive factors):

5 = critical; if present usually guarantee selecting the applicant; 4 = important; if present is very useful in selecting the applicant; 3 = not very important, but useful if present; 2 = rarely considered when selecting residents; 1 = not important at all when selecting residents.

Scoring scale (negative factors):

5 = critically concerning; if present usually guarantee rejecting the applicant; 4 = very concerning; if present is likely used to reject the applicant; 3 = not very concerning; may be considered to reject applicants if there is a strong competition between applicants; 2 = rarely considered as concerning; rarely used to reject applicants; 1 = not a concern; not used at all in rejecting applicants.

**Table 5:** Average score* of criteria that may affect the acceptance of an applicant to Obstetrics and Gynecology and Surgical residency programs in Kuwait, 2011

**Criteria**	**Anesthesia**	**General Surgery**	**Obstetrics & Gynecology**	**Ophthalmology**	**Orthopedic Surgery**	**Otorhinolaryngology (Head & Neck Surgery)**	**Urology**
	**N = 6**	**N = 6**	**N = 6**	**N = 3**	**N = 4**	**N = 4**	**N = 3**
Positive factors							
Gender	2.00	2.33	2.66	1.33	2.75	3.25	1.33
Nationality	3.83	4.50	4.00	3.00	3.25	4.50	5.00
Grade Point Average (GPA)	4.16	4.16	4.33	4.00	3.25	4.25	3.33
Honors during medical school	3.83	4.00	4.33	4.00	3.50	4.25	3.33
Rank on class during medical school	3.83	3.33	4.16	3.66	2.50	3.75	3.00
Grades in required clerkships	3.66	3.50	4.00	3.66	3.75	4.25	4.00
Grades in clinical courses	3.66	3.33	4.33	3.00	3.75	3.50	3.66
Grades in pre-clinical courses	3.50	2.83	3.33	2.66	3.50	3.25	2.00
Electives in required clerkships	4.16	3.00	3.66	3.33	4.25	5.00	4.33
Research experience	3.50	3.33	3.50	3.66	3.75	5.00	2.33
Publications in indexed journals	3.33	3.33	3.16	3.66	4.00	5.00	2.00
Recommendation letters	4.16	3.50	3.33	3.00	2.75	3.50	3.66
Attending scientific conferences during medical school	3.66	3.16	3.33	3.00	3.00	3.00	2.00
Medical school reputation	4.66	3.50	4.16	3.33	4.00	3.25	3.00
Meaningful involvement in extracurricular activities during medical school	3.50	2.83	3.50	2.33	3.50	3.00	2.33
Interview performance	4.66	4.83	4.66	4.66	5.00	4.75	4.33
Negative factors							
Received disciplinary action in medical school	4.83	4.33	4.66	3.66	4.50	4.25	3.66
Received failure in a required clerkship	3.66	3.33	4.16	3.66	4.00	4.50	2.66
Took extended time to graduate for academic reasons	3.83	3.83	4.33	4.00	3.25	4.00	3.00
Graduated in the lower third of class	3.00	3.50	3.83	4.00	2.75	3.50	2.66
Received a failure in a preclinical course	3.66	2.67	2.66	3.00	3.00	3.25	2.00
Had family responsibilities	3.16	1.50	2.66	3.66	2.50	2.50	2.00
Spent a long period after internship to apply for a residency program	3.50	2.66	3.16	3.66	2.75	4.25	3.00

*Scoring scale (positive factors):

5 = critical; if present usually guarantee selecting the applicant; 4 = important; if present is very useful in selecting the applicant; 3 = not very important, but useful if present; 2 = rarely considered when selecting residents; 1 = not important at all when selecting residents.

Scoring scale (negative factors):

5 = critically concerning; if present usually guarantee rejecting the applicant; 4 = very concerning; if present is likely used to reject the applicant; 3 = not very concerning; may be considered to reject applicants if there is a strong competition between applicants; 2 = rarely considered as concerning; rarely used to reject applicants; 1 = not a concern; not used at all in rejecting applicants.

**Table 6:** Average score* of criteria that may affect the acceptance of an applicant to Laboratory Medicine, Nuclear Medicine and Radiology residency programs in Kuwait, 2011

**Criteria**	**Laboratory-Clinical Biochemistry & Metabolism**	**Laboratory-Clinical Hematology**	**Laboratory-Clinical Microbiology**	**Laboratory-Clinical Virology**	**Laboratory-Diagnostic Immunology**	**Laboratory-(Histo & Cyto) Pathology**	**Nuclear Medicine**	**Radiology**
	**N = 4**	**N = 5**	**N = 8**	**N = 3**	**N = 4**	**N = 3**	**N = 5**	**N = 4**
Positive factors								
Gender	1.25	1.00	1.12	2.00	1.00	1.33	1.00	1.50
Nationality	2.25	3.40	3.12	4.33	2.25	1.66	4.80	2.75
Grade Point Average (GPA)	3.75	3.60	3.75	4.00	3.75	3.66	4.40	4.50
Honors during medical school	3.75	3.80	3.25	3.66	3.75	3.66	3.80	4.50
Rank on class during medical school	3.00	3.40	3.12	3.33	3.50	3.66	3.40	4.00
Grades in required clerkships	4.00	3.80	2.50	2.66	3.75	3.66	3.00	3.50
Grades in clinical courses	4.00	3.60	2.62	2.66	3.50	3.33	3.00	3.50
Grades in pre-clinical courses	3.75	3.60	2.62	2.66	3.25	3.66	3.00	2.50
Electives in required clerkships	3.50	3.60	2.62	2.66	3.75	3.33	3.20	4.50
Research experience	4.00	3.80	3.00	3.00	3.50	4.33	3.00	3.25
Publications in indexed journals	4.00	3.80	3.00	3.00	3.25	3.66	3.20	2.75
Recommendation letters	3.00	3.20	3.00	3.33	3.50	3.33	4.00	3.25
Attending scientific conferences during medical school	3.25	3.40	2.87	2.66	3.75	3.66	3.20	3.00
Medical school reputation	3.00	3.60	2.62	3.00	3.50	2.00	2.80	4.25
Meaningful involvement in extracurricular activities during medical school	2.50	2.80	2.12	2.66	3.50	3.33	3.00	3.25
Interview performance	4.50	4.80	4.50	4.33	4.25	4.66	4.80	5.00
Negative factors								
Received disciplinary action in medical school	3.50	3.00	2.37	4.33	4.00	3.66	4.20	4.75
Received failure in a required clerkship	3.00	4.00	2.87	3.66	4.25	3.33	4.00	3.00
Took extended time to graduate for academic reasons	3.50	3.40	3.37	3.33	4.00	3.33	3.00	3.50
Graduated in the lower third of class	3.00	3.00	2.25	3.33	2.75	3.33	3.20	3.25
Received a failure in a preclinical course	3.00	3.00	2.37	3.00	3.75	3.00	2.60	3.00
Had family responsibilities	2.25	2.00	1.87	2.66	2.25	2.66	1.80	1.50
Spent a long period after internship to apply for a residency program	3.00	2.80	2.75	3.66	3.25	2.33	3.00	4.50
Did not participate in extracurricular activities during medical school	2.75	1.80	1.87	2.33	3.00	2.33	2.40	2.50

*Scoring scale (positive factors):

5 = critical; if present usually guarantee selecting the applicant; 4 = important; if present is very useful in selecting the applicant; 3 = not very important, but useful if present; 2 = rarely considered when selecting residents; 1 = not important at all when selecting residents.

Scoring scale (negative factors):

5 = critically concerning; if present usually guarantee rejecting the applicant; 4 = very concerning; if present is likely used to reject the applicant; 3 = not very concerning; may be considered to reject applicants if there is a strong competition between applicants; 2 = rarely considered as concerning; rarely used to reject applicants; 1 = not a concern; not used at all in rejecting applicants.

Participants' comments are shown in Additional file 1. A total of 41 comments were documented. Most of the comments (26) were about the gender and the nationality of the applicants. Eight participants reported a preference of male residents because of multiple reasons such as a lack of male specialists in their specialty. In addition, 16 comments about the nationality of the applicant were received. These comments indicate that, when selecting residents, the priority is for Kuwaiti applicants. Other comments about GPA, honors during medical school, grades in required clerkship, research experience, publications, recommendation letters, extracurricular activities, interview performance and receiving disciplinary actions during medical school are also shown in appendix 1.

## Discussion

The results of this study revealed that getting accepted in a residency program in Kuwait is competitive. In order to increase the likelihood of their acceptance, students and junior doctors should work hard on developing their curriculum vitae (CV) using this paper as a guide.

It was noted that members of residency programs in Kuwait were looking for a diverse population of residents by accepting more males and Kuwaiti applicants. Diversity was found to be an important aspect of health care [17]. By enrolling more residents according to their sociodemographic background in residency programs, diversity can be achieved and health care may improve.

Like others, interview performance appeared to be the most important criterion used to select residents for residency programs in Kuwait [2,11,14]. Interview styles vary from program to another in aspects of duration, number of interviewers, guidelines and assignments [11]. Some programs utilize the interview to know the applicant's background and interests, while others use it to assess the applicant's ability to answer ethical questions, perform clinical skills and perform manual dexterity skills, as well as to evaluate the applicant by a psychologist/psychiatrist [4,11,18]. In addition, Brothers and Wetherholt (2007) revealed that faculty evaluation of applicant's personal characteristics and reference letter quality during surgical residency interviews was likely to predict subsequent clinical performance of the resident [15].

In Kuwait, criteria that reflect academic performance and interest in the required specialty (GPA, honors during medical school, rank on class, electives and grades in required clerkships, clinical and pre-clinical courses) were usually considered important when selecting residents. Similar criteria received similar weight in the USA where grades in required clerkship was ranked as the most important criterion used for selecting residents, followed by other academic criteria such as number of honors grades, class rank and USMLE scores [1,2,10]. USMLE scores (which reflect academic performance) were found to be positively associated with in-training examination score in some residency programs [19-21]. However, criteria that reflect medical school performance did not correlate with Obstetrics & Gynecology residents' performance in Johns Hopkins Medical Institutions [16].

Medical school reputation followed the academic criteria in its importance level for selecting residents in Kuwait. Our participants did not provide reasons for this; however, past experiences with residents who graduated from different medical schools might result in preferring some universities' graduates than others. Likewise, most programs directors in the USA considered the applicant's medical school reputation as an important criterion of selecting residents [1,2].

During residency training, residents are required to conduct research and publish papers or posters. In our study, the applicant's research experience and publications in indexed journals were ranked high across most of the programs. For example, a score of 5.00/5.00 was given to these criteria by members of the Otorhinolaryngology residency program. In the UK, having an additional research degree was considered the second most important criterion in assessing applicants for residency training [3]. On the contrary, these criteria were ranked very low in the USA; however, it was ranked high by directors of the competitive programs [1,2].

Recommendation letters were found to be useful in selecting residents in our study. In the USA, a high value was placed on recommendation letters by residency programs' directors [1,2]. More than 90% of the directors said that they require at least two letters of recommendation before considering a student for interview [2].

Despite students' perceptions that attending scientific conferences during medical school is an important component of their CV, our findings indicate that it ranks low among other criteria used to select residents in Kuwait. This low rank might be because conferences usually provide knowledge of an advanced level, and they are designed for postgraduate education.

Most medical students spend a substantial time practicing extracurricular activities. When evaluated by members of residency programs in Kuwait, these activities received a low rank among the criteria that are used to select residents. However, some participants believed that extracurricular activities reflect leadership skills, maturity and professionalism of the applicant. This criterion received low rank also in other studies [11,22,23].

Receiving disciplinary action in medical school was ranked as the most concerning criterion that might be used to reject applicants in Kuwait. One participant mentioned that cheating in exams will automatically disqualify the candidate from getting accepted in his/her program. In the USA, this criterion also received the higher score among other criteria that are used to reject applicants [2].

Both in Kuwait (our study) and USA, having family responsibilities was not found to be concerning when selecting residents [2]. However, it was noted that married residents performed better than unmarried residents in orthopaedic residency training examinations [24].

Although this study provided useful information about the criteria of selecting residents in Kuwait, it has limitations. The participants rated the criteria we proposed in our questionnaire; however, we are not sure if they used these criteria in selecting applicants before participating in this study. Moreover, we did not have data that can demonstrate how these criteria correlate with the residents' performance during their training program. Also, for some criteria that we found to be useful in selecting residents, there is a need for future studies to explore these criteria in details; for example, studying the process and components of the interview, knowing what are the residents' selection committee looking for in the recommendation letters, and what extracurricular activities would be useful to improve the applicants CV.

## Conclusions

In our study, we provided important information about the criteria that are used to select or reject residents for residency programs in Kuwait. These information can be used be medical students and junior doctors who are interested in completing their postgraduate training in Kuwait. Also, mentors and supervisors can use this study to improve the chances of their students in getting accepted in their preferred residency program.

We suggest future studies that aim to investigate the details of each criterion individually. Also, we recommend prospective studies to assess the relationship of the criteria that are used to select residents and the success during residency training.

## Competing interests

The authors declare that they have no competing interests.

## Authors’ contributions

All authors participated in planning, conducting, analyzing and writing the study equally. All authors read and approved the final manuscript.

## Pre-publication history

The pre-publication history for this paper can be accessed here:

http://www.biomedcentral.com/1472-6920/13/4/prepub

## Supplementary Material

Additional file 1 **Appendix 1. **Residency programs' members free comments on criteria that may affect the selection of residents for residency programs in Kuwait, 2011. Click here for file
