# Supplementary material for: Selection criteria of residents for residency programs in Kuwait
Source: BMC Med Educ. 2013 Jan 19;13:4. doi: 10.1186/1472-6920-13-4 (PMC3552967; doi:10.1186/1472-6920-13-4)
Supplement: Additional file 1 — Appendix 1. Residency programs' members free comments on criteria that may affect the selection of residents for residency programs in Kuwait, 2011. [file 1472-6920-13-4-S1.doc]

| Criteria  Appendix 1. Residency programs' free comments on criteria that may affect the selection of residents for residency programs in Kuwait, 2011 | Quotations |
| --- | --- |
| Positive factors |  |
| Gender | 1. Prefer male residents [Radiology, Otorhinolaryngology, Orthopaedics, Anesthesia, Primary Health Care x2] 2. Prefer male because Orthopaedic specialty needs physical fitness [Orthopaedics] 3. In Dermatology, females are more. So we are trying to select more males recently [Dermatology] 4. No impact on selection [Internal Medicine, Clinical Microbiology] |
| Nationality | 1. Prefer Kuwaiti residents [Orthopaedics x2, Anesthesia x2, Dermatology, Clinical Biochemistry and Metabolism, Clinical Virology, Otorhinolaryngology, Physical Medicine and Rehabilitation, Clinical Microbiology, Clinical Hematology, Primary Health Care x2] 2. Kuwaiti > applicants from the Gulf Cooperation Council (GCC) countries > Others [Nuclear Medicine] 3. I prefer Kuwaitis due to shortage of Kuwaitis in Clinical Microbiology [Clinical Microbiology] 4. I prefer Kuwaitis because non-Kuwaitis usually do not stay for a long time in Kuwait after completing their training [Clinical Microbiology] |
| Grade Point Average (GPA) | 1. Gives good support for the program [Anesthesia] 2. Important because it reflects the candidate's academic performance [Internal Medicine] 3. GPA reflects motivation of possibility of completing the program successfully [Clinical Hematology] 4. Higher GPA reflects hard working student with higher academic achievements [Diagnostic Immunology] |
| Honors during medical school | 1. Excellent candidates make excellent program [Anesthesia] |
| Grades in required clerkships | 1. Good grades in required specialty-related courses indicate interest in the field [Clinical Hematology] |
| Research experience | 1. Shows interest in academic aspects [Internal Medicine] |
| Publications in indexed journals | 1. Extremely important [Clinical Biochemistry and Metabolism] |
| Recommendation letters | 1. Prefer direct communication [General Surgery, Pediatrics] |
| Meaningful involvement in extracurricular activities during medical school | 1. This shows leadership skills [Clinical Microbiology] 2. Reflects maturity and professionalism [Diagnostic Immunology] |
| Interview performance | 1. Extremely important [Clinical Microbiology] |
| Rank on class during medical school, grades in clinical courses, grades in pre-clinical courses, electives in required clerkships, attending scientific conferences during medical school, and medical school reputation | No comments |
| Negative factors |  |
| Received disciplinary action in medical school | 1. History of cheating in exams automatically disqualify the candidate [Clinical Biochemistry and Metabolism] 2. This will highly affect the acceptance of the applicant [Physical Medicine and Rehabilitation] |
| Received failure in a required clerkship, took extended time to graduate for academic reasons, graduated in the lower third of class, received a failure in a preclinical course, had family responsibilities, spent a long period after internship to apply for a residency program, and did not participate in extracurricular activities during medical school | No comments |
